# Neural correlates of early cognitive dysfunction in Parkinson's disease

**DOI:** 10.1002/acn3.767

**Published:** 2019-03-28

**Authors:** Rimona S. Weil, Joel S. Winston, Louise‐Ann Leyland, Katerina Pappa, Ribeya B. Mahmood, Huw R. Morris, Geraint Rees

**Affiliations:** ^1^ Dementia Research Centre UCL London United Kingdom; ^2^ Wellcome Centre for Human Neuroimaging UCL London United Kingdom; ^3^ National Hospital for Neurology and Neurosurgery London United Kingdom; ^4^ Institute of Cognitive Neuroscience UCL London United Kingdom; ^5^ Department of Clinical and Motor Neuroscience UCL Queen Square Institute of Neurology London United Kingdom; ^6^ Movement Disorders Centre UCL Queen Square Institute of Neurology London United Kingdom

## Abstract

**Objective:**

Dementia is a common and feared aspect of Parkinson's disease but there are no robust predictors of cognitive outcome. Visuoperceptual deficits are linked to risk of dementia in Parkinson's disease but whether they predict cognitive change is not known, and the neural substrates of visuoperceptual dysfunction in Parkinson's have not yet been identified.

**Methods:**

We compared patients with Parkinson's disease and unaffected controls who underwent BOLD fMRI while performing our previously validated visuoperceptual task and tested how functional connectivity between task‐specific regions and the rest of the brain differed between patients who performed well and poorly in the task.

**Results:**

We show that task performance at baseline predicts change in cognition in Parkinson's disease after 1 year. Our task‐based fMRI study showed that the performance in this task is associated with activity in the posterior cingulate cortex/precuneus. We found that functional connectivity between this region and dorsomedial prefrontal cortex was reduced in poor performers compared with good performers of this task.

**Interpretation:**

Our findings suggest that functional connectivity is reduced between posterior and anterior hubs of the default mode network in Parkinson's patients who are likely to progress to worsening cognitive dysfunction. Our work implicates posterior default mode nodes and their connections as key brain regions in early stages of dementia in Parkinson's disease.

## Introduction

Dementia is one of the most debilitating aspects of Parkinson's disease, affecting 50% of people within 10 years of diagnosis, with wide variability in timing and severity.^1^ Being able to determine the anatomical basis of the earliest stages of Parkinson's dementia is a priority to enrich populations for clinical trials of treatments that slow progression of Parkinson's dementia. Identifying neuroanatomical substrates of Parkinson's dementia will also provide important insights into the mechanistic basis of selective vulnerability.

The anatomical substrates of the earliest stages of Parkinson's dementia are poorly defined and to date, neuroimaging predictors of Parkinson's dementia have been elusive. Structural measurements of gray matter atrophy using conventional techniques show inconsistent patterns,^2^, ^3^, ^4^ most likely because cell death, indexed by gray matter atrophy, is a late event in Parkinson's dementia.^5^ Functional changes linked with tests predictive of cognitive dysfunction may be better suited to detect the earliest signs of cognitive involvement in Parkinson's disease.^6^

Recent evidence suggests that Parkinson's patients with visual processing deficits are at higher risk of dementia. In population studies, patients making errors copying intersecting pentagons are at double the risk of dementia at follow‐up.^1^ Patients with occipital hypometabolism at baseline show higher rates of converting to Parkinson's dementia,^7^ and in postmortem studies, Parkinson's patients with an occipital distribution of Lewy‐related pathology developed more rapid dementia and died sooner.^8^ One event‐related fMRI study examined brain activity during visuoperceptual tasks and showed differences in brain activity in superior parietal regions, in the absence of differences in task accuracy,^9^ but how this relates to development of Parkinson's dementia has not been shown.

Emerging studies of functional connectivity in cognitively intact Parkinson's suggest that changes in the default mode network (DMN) may be linked to cognitive performance in non‐demented Parkinson's disease.^10^, ^11^ However, how this relates to development of dementia in Parkinson's disease is not known.

We recently developed a sensitive test of visuoperceptual processing, and showed that the performance in this test is related to an independent risk score for Parkinson's dementia.^12^ The neural correlates of performing this visuoperceptual task are not known, and whether this task can predict Parkinson's dementia has not yet been shown. Here, we measured BOLD signals while people with Parkinson's and age‐matched controls performed our visuoperceptual task. We hypothesized that (1) People performing worse in this task would show worse cognitive performance after 1 year. (2) Higher‐order visual processing regions would be implicated in this task in unaffected controls. (3) These regions would show reduced activity in Parkinson's patients that performed poorly on our task, compared with those that perform well. (4) Task‐dependent functional connectivity would be reduced in poorly performing Parkinson's patients compared with high performers.

## Methods and Materials

### Participants

Twenty people with Parkinson's disease were recruited from our UK center between September 2015 and May 2016. Inclusion criteria were early to mid‐stage Parkinson's disease (Queen Square Brain Bank Criteria). Exclusion criteria were confounding neurological disorders, dementia, and metallic implants considered unsafe for MRI scanning (e.g., permanent pacemakers). Participants continued their usual therapy and Levodopa equivalent daily dose (LEDD) was calculated.^13^ One participant missed an excessive number of trials (>40% in each experimental run) and was excluded. The data reported here therefore include 19 people with Parkinson's disease. Ten age‐matched controls were recruited from university databases and unaffected spouses. All participants gave written informed consent and the study was approved by the Queen Square Research Ethics Committee.

### Clinical evaluation

Severity of symptoms was assessed using the MDS‐UPDRS. Cognition was assessed using the Montreal Cognitive Assessment (MoCA), at baseline and at follow‐up (mean 12.6 months, range 8–17 months), in 22 participants (15 with Parkinson's). Change in cognitive performance was quantified as the difference between follow‐up and baseline scores. Visual acuity was assessed using a 6‐m Snellen chart and converted to decimal acuity.^14^ Contrast sensitivity was measured using a Pelli‐Robson chart (SSV‐281‐PC) (http://www.sussex-vision.co.uk) (Table 1).

**Table 1:** Demographics of participants

	Controls Mean (SD)	PD Mean (SD)	*T* (or *χ* ^2^) (df)	*P*
*n*	10	19	–	–
Age (SD), (range)	64.8 (11.2), (45–78)	64.2 (6.1), (55–72)	0.15 (11.9)	0.88
M/F	4/6	10/9	0.4 (1)	0.52
Disease duration PD (years)	NA	5.2 (3.5)	–	–
H&Y	NA	1.4 (0.60)	–	–
MDS‐UPDRS	4.1 (4.3)	27.9 (11.7)	−7.8 (25)	<0.0001*
LEDD	NA	643.0 (372)	–	–
Best visual acuity	1.06 (0.2)	1.01 (0.2)	0.56 (20)	0.58
Contrast sensitivity (both eyes)^1^	1.82 (0.14)	1.82 (0.19)	–0.11 (21)	0.91
MOCA (baseline)	28.9 (1.6)	28.9 (1.2)	0.009 (14.5)	0.99
MOCA (follow‐up) (*n*)	28.4 (1.5) (7)	28.3 (1.5) (9)	0.13 (13)	0.90
Time to MOCA follow‐up (Months)	12.4 (3.8)	12.6 (2.9)	−0.15 (9)	0.89

	High performers with Parkinson's	Low performers with Parkinson's	*T* (or *χ* ^2^) (df)	*P*
*n*	11	8		
Age	62.4 (5.9), (55–70)	66.6 (5.9), (56–72)	−1.5 (15)	0.15
M/F	3/8	7/1	6.7 (1)	0.0094*
Disease duration (years)	3.8 (3.3)	7.2 (3.2)	−2.3 (16)	0.039*
H&Y	1.1 (0.3)	1.8 (0.7)	−2.5 (9)	0.036*
MDS‐UPDRS	22.6 (9.4)	35.1 (11.1)	−2.6 (14)	0.023*
LEDD	488 (259)	857 (430)	−2.1 (11)	0.055
Best visual acuity	1.05 (0.2)	0.96 (0.2)	0.91 (15)	0.38
Contrast sensitivity (both eyes)	1.87 (0.16)	1.76 (0.22)	1.2 (12)	0.27
MOCA (baseline)	28.7 (1.6)	29.1 (0.4)	−0.82 (11)	0.43
MOCA (follow‐up) (*n*)	28.7 (1.5) (9)	27.8 (1.5) (6)	1.6 (11)	0.31
Time to MOCA follow‐up (Months)	11.9 (2.7)	13.0 (3.6)	−0.56 (5)	0.60

Df, degrees of freedom; H&Y, Hoehn and Yahr; LEDD, Levodopa equivalent dose; MDS‐UPDRS, movement disorder society unified Parkinson's disease rating scale; MOCA, Montreal cognitive assessment; PD, Parkinson's disease; SD, standard deviation.

^1^ Data from one control participant not available.

* *P* < 0.05.

### Experimental task

Stimuli were generated as previously described.^12^ Briefly, images of 1000 cats and dogs from an online database (http://www.kaggle.com) were cropped and converted to grayscale. Fourier transforms of each image were computed to produce magnitude and phase images. The phase matrix of each cat or dog image was skewed along the *x*‐axis by a variable amount of skew (four levels: 0, 1.4, 2.2, and 2.8 a.u.). This was combined with a proportion of white noise and recombined with the average magnitude matrix of the series. Resulting images had identical spatial frequency with four levels of skew. Skew levels were chosen based on psychophysical thresholds measured previously, to include no skew, two moderate levels, that equated to median levels of tolerated skew in people with Parkinson's and controls, respectively, and extreme skew not tolerated by any participants.^12^

Control images were generated in the same way, but instead of an affine transformation, a varying proportion of visual noise was added (four levels: 0, 0.5, 0.8, and 1.2 a.u.). The amount of noise varied according to the following formula:

Test image=Image∗(1−Contrast level)+(Noise matrix∗Contrast level)

Stimuli were presented in MATLAB 2014a (MathWorks Inc, Natick, MA) using Cogent 2000 (http://www.vislab.ucl.ac.uk/cogent_2000.php) onto an Epson EH‐TW5900/59100 projector (screen width 26 cm, screen height 21 cm, at approximately 78 cm viewing distance). Participants viewed the screen through a mirror attached to the head coil. Image widths subtended 32.5 × 8.7 degrees visual angle with mean luminance 6.48 cd/m^2^.

**Figure 1:**
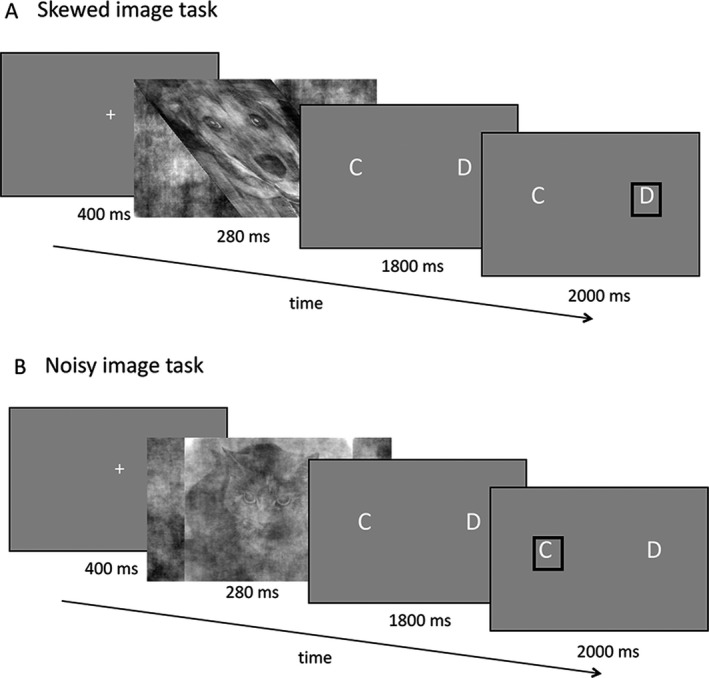
Experimental task. (A) Each trial started with a plain gray screen with a central fixation cross for 400 msec. On each trial, a different image of a cat or dog was shown that was skewed by a variable amount (four levels of skew between 0 a.u. and 2.8 a.u., defined in pilot sessions), order pseudorandomized. The image was shown for 280 msec. This was followed up by a screen with the letters C and D, indicating cat and dog. Participants indicated whether they had seen a cat or a dog by pressing a key on a response pad whilst inside the scanner. (B) Control trials with noisy images were identical in structure, but increasing amounts of visual noise were added (four levels of noise: between 0 and 1.2 a.u., defined in pilot sessions).

All participants underwent practice sessions outside the scanner, immediately prior to image acquisition to ensure familiarity with the task. Each trial consisted of a fixation cross for 400 msec, followed by the skewed or noisy image for 280 msec (Fig. 1). Short presentation times were used to avoid confounds from eye movements. Participants responded using a fiber‐optic response pad, with side of response pseudorandomized at the start of every run, but kept constant for the run duration. This avoided a laterality bias for responses and minimized confusion between trials. Response time window was 1800 msec. Intertrial interval was jittered with mean 450 msec. There were six experimental task runs, each lasting approximately 6 min: four runs of skewed images and two runs of noisy images, with order of skewed and noisy runs randomized for each participant.

### Imaging acquisition

Participants were scanned (at baseline) in a Siemens Trio 3‐Tesla MRI scanner with 32‐channel head coil. Functional data were acquired with a 2D gradient‐echo planar sequence: 48 transverse slices, slice thickness = 2.5 mm, gap between slices = 0.5 mm, repetition time TR = 3.36 sec, TE 30 msec, and inplane resolution 3.0 × 3.0 × 3.0 m. The first five volumes were discarded to allow T1 equilibration.

A B0 field map was obtained after functional data acquisition: short TE = 10 msec; long TE = 12.46 msec; polarity of phase‐encode blips = −1; total EPI readout time = 37 msec, ascending slice order. Heart rate and respiration were monitored using an MRI‐compatible pulse oximeter (Nonin 8600 FO) and pneumatic belt,^15^ and recorded, along with scanner pulses via a Cambridge Electronic Devices Micro 1401 Mk11 connected to a laptop running Spike2 version 6. A T1‐weighted structural scan was acquired for each participant and used for normalization of functional data (TR = 7.92 msec, TE = 2.45 msec, T1 = 910 msec, flip angle *α* = 16°, 176 = slices, 1 × 1 × 1 mm voxels, FIV = 256 × 240 mm^2^ 16).

### Behavioral analyses

For each level of skew or noise, we calculated *d* prime (*d*′), using the following equation:

$$d^{\prime }=z\left(H\right)-z\left(\text{FA}\right)$$

where *z* indicated inverse of the cumulative normal distribution, H the hit rate, and FA the false alarm rate. Extreme values were corrected by dividing 0.5 by number of trials at that level.^17^ We checked the number of missed trials per run, and excluded runs where missed trials exceeded 40% (1 participant, 1 run). As described above, one more participant was excluded from analyses, due to excessive missed trials.

To categorize participants as low or high performing, we analyzed the performance at the second skew level for all odd trials. Participants with *d*′ ≥ 1 were considered high performers. For subsequent behavioral analyses at that level, we only included even trials, to avoid resampling the same dataset. Between‐group differences in response times, performance, and clinical and demographic measures were assessed using ANOVAs. Post hoc *t‐*tests were used to compare the groups. Between‐group differences in categorical variables were assessed using Chi‐squared contingency tests.

### Imaging analysis

Data analysis used SPM12(WTCN; http://www.fil.ion.ucl.ac.uk/spm) and involved standard methods (realignment and unwarping, normalization) using parameters estimated from normalization of segmented structural images that were coregistered to EPIs and smoothing with an 8‐mm isotropic Gaussian). We performed statistical inferences using the generalized linear model (GLM), implemented in SPM12. Events were characterized by stick functions at time of onset convolved with the canonical hemodynamic response function to provide regressors for the GLM. Presentation of images was taken as onset times. Nine onset types were modeled: eight for each level of skewed (0, 1.4, 2.2, and 2.8 a.u.) or noisy image (0, 0.5, 0.8, and 1.2 a.u.), one for missed trials.

The six realignment parameters estimated during preprocessing were included as estimates of movement and cardiac and respiratory contributions to the fMRI noise were modeled.^18^, ^19^ Altogether the full physiological noise model yielded a set of 20 regressors that were included in the GLM for each block. A block‐specific mean was also included in the GLM.

Statistical inference was at the random effects level. Maps of contrasts of parameter estimates from the single‐subject GLMs formed the raw data for inference in a second‐level analysis where subjects were treated as random effects. Second‐level analysis was initially restricted to control participants, to determine regions maximally involved in conditions of interest during normal visual processing.

We extracted parameter estimates within the whole main cluster for high and low performers for each of the eight simple effects (four levels of difficulty for skew and four levels of difficulty for the noise task). We hypothesized that high performers would show parameter estimates similar to those seen in unaffected controls, whereas low performers would show differences in the pattern of parameter estimates.

### Psychophysiological interactions

We reasoned that regions showing differences in task‐specific functional activity would relate to well‐defined brain networks. Consequently, we examined functional connectivity between the posterior cingulate cortex (PCC)/precuneus and the rest of the brain during the performance of the visuoperceptual task using a psychophysiological interaction (PPI) analysis.^20^, ^21^ We set up a GLM with regressors capturing the physiological effect (time series for an 8‐mm sphere centered on peak voxel of the PCC/precuneus cluster [−3, −64, 19] derived from the whole brain analysis in unaffected participants), the psychological contrast of interest, and the psychophysiological interaction term (i.e., physiological effect × psychological contrast of interest). The GLM included six motion parameters and 20 cardiac and respiratory regressors to correct for these sources of noise. These formed the new raw data for inference in a second‐level analysis. At the second level, we compared functional connectivity between the PCC/precuneus and the rest of the brain between high‐ and low‐performing participants with Parkinson's disease. As this analysis was considered exploratory, we accepted a lower threshold of significance, *P *<* *0.001 uncorrected.

## Results

### Demographics

Twenty Parkinson's patients and 10 unaffected controls completed the study. One participant with Parkinson's was removed due to excessive missed trials (see Methods), leaving 19 Parkinson's patients and 10 controls in the analyses here. Participants were well‐matched for age and gender. The mean age of participants with Parkinson's disease was 64.2 (±6.1) years, and mean age of controls was 64.8 (±11.2) years (Table 1). The mean disease duration was 5.2 ± 3.5 years, and the mean Hoehn and Yahr (H&Y) score was 1.4 (±0.6). There were no significant differences between the groups in measures of cognition, visual acuity, or contrast sensitivity (Table 1), and none of our participants had dementia.

### Behavioral performance

As expected, all participants’ performance worsened as images became more skewed and more noisy, with a main effect of difficulty for both the skew (*F*(3,81) = 88.2, *P *<* *0.0001) and noise task (*F*(3,81) = 101.1, *P *<* *0.0001). There was no main effect of Parkinson's disease, or interaction between the presence of Parkinson's disease and the level of difficulty for the skewed or noise tasks.

We used performance measured by *d*′ ≥ 1 at the second level of skew (1.4 a.u.) to divide Parkinson's patients into high and low performers. This level was used as our previous work^12^ showed this was the mean threshold of tolerance in Parkinson's disease. This generated two groups: 8 low performers and 11 high performers. These groups did not differ in cognitive performance or visual acuity and contrast sensitivity. However, there was a significantly higher proportion of men in the poor‐performing group (7/8, compared with 3/11, *P *=* *0.009). Disease duration was higher in the poor‐performing group, as were measures related to disease severity including H&Y, UPDRS, and levodopa dose (Table 1).

The performance in the skew task showed a main effect of group (high vs. low performers): *F*(1,17) = 35.5, *P *<* *0.0001); a main effect of difficulty: *F*(3,51) = 58.1, *P *<* *0.0001; but there was no interaction between the group and amount of skew: *F*(3,51) = 2.2, *P *=* *0.10) (Table 2). This result was not surprising, as we had divided participants into high and low performers based on performance in this task. This division was performed for later analysis of the fMRI data based on the performance level. We include it here so that demographics and range of performance in the two groups can be seen.

**Table 2:** Performance in skew and visual noise tasks

Level	Performance in controls *d*′	PD high performers *d*′	PD low performers *d*′	*T* (df) (controls vs. PD high)	*P*	*T* (df) (controls vs. PD low)	*P*	*T* (df) (PD high versus low)	*P*
Skew task									
Skew 1	2.12 (0.37)	2.30 (0.22)	1.51 (0.46)	−1.4 (15)	0.17	3.0 (13)	0.010^a^	4.48 (9)	0.0014^a^
Skew 2	1.60 (0.56)	1.52 (0.31)	0.59 (0.64)	0.39 (14)	0.70	3.51 (14)	0.0035^a^	3.8 (9)	0.0039^a^
Skew 3	0.70 (0.5)	0.82 (0.50)	0.33 (0.40)	−0.56 (19)	0.58	1.7 (16)	0.10	2.4 (17)	0.028^a^
Skew 4	0.61 (0.41)	0.65 (0.38)	0.29 (0.24)	−0.22 (18)	0.82	2.0 (15)	0.058^a^	2.5 (17)	0.022^a^
Visual noise task									
Noise 1	1.94 (0.36)	2.07 (0.42)	1.56 (0.46)	−0.74 (19)	0.47	1.9 (13)	0.073	2.5 (14)	0.025^a^
Noise 2	1.22 (0.55)	1.24 (0.51)	0.44 (0.52)	−0.09 (18)	0.94	3.1 (16)	0.0072^a^	3.4 (15)	0.0042^a^
Noise 3	0.082 (0.57)	−0.056 (0.27)	−0.18 (0.66)	0.70 (13)	0.50	0.89 (14)	0.39	0.50 (9)	0.63
Noise 4	−0.013 (0.53)	0.067 (0.33)	−0.066 (0.31)	−0.41 (15)	0.69	0.27 (15)	0.79	0.90 (16)	0.38

Performance at each level of skew and each level of the blur task, in each of the three groups, measured using d prime: unaffected controls, and Parkinson's participants in the high‐ and low‐performance groups. Note that groups were defined by performance at the second skew level (Skew 2), using alternate (odd‐numbered) trials. Data at the second level of skew shown here are therefore for even trials.

Df, degrees of freedom; PD, Parkinson's disease; vs, versus.

a *P* < 0.05.

For the visual noise task, we found a main effect of group (high vs. low performers): *F*(1,17) = 24.8, *P *=* *0.00011; main effect of difficulty: *F*(1,55) = 119.6, *P *<* *0.0001; but the interaction between the group and amount of noise did not reach significance: *F*(1,55) = 2.3, *P *=* *0.14).

A three‐way ANOVA of the group (three levels: controls, high, and low performers), difficulty (four levels), and task (two levels: skew vs. noise) showed a main effect of group *F*(1,17) = 44.2, *P* < 0.001; main effect of difficulty, *F*(3,119) = 137.4, *P *<* *0.0001; and main effect of task type, *F*(1,119) = 33.1, *P *<* *0.0001. There was an interaction between the group and difficulty, *F*(1,17) = 4.6, *P *=* *0.0042; trend to interaction between the group and task type, *F*(1,119) = 3.3, *P *=* *0.070; an interaction between difficulty and task type, *F*(3,119) = 4.1, *P *=* *0.0084. However, there was no interaction between the group, task, and difficulty.

The interaction of difficulty and task and trend toward the group (high performers vs. low performers) and task (skew vs. noise) suggests some specificity to type of task. Planned post hoc *t*‐tests show this is driven by difference between high and low Parkinson's performers in higher levels of difficulty in the skew task, but not in the visual noise task (Table 2).

### Visual performance predicts cognitive change after 1 year

**Figure 2:**
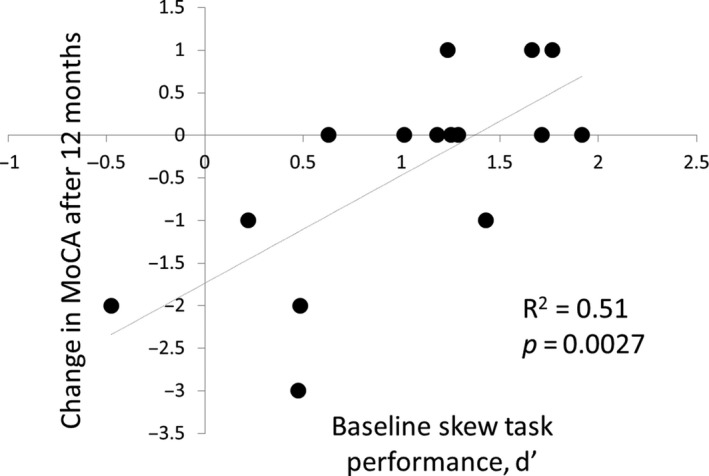
Relationship between baseline performance in the skew task and change in MoCA after 12‐month follow‐up.

We found a strong association between performance in the skew task and change in cognitive performance after 1 year in Parkinson's patients, *R* ^2^=0.51, *F*(1,13) = 13.6, *P *=* *0.0027 (Fig. 2). This effect was also seen when we included unaffected participants (*R* ^2^ = 0.23, *F*(1,20) = 6.1, *P *=* *0.023), although with a lower value for *R* ^2^, suggesting this relationship is more specific to predict cognitive change in Parkinson's disease. We also found a strong association between performance in the noise task and change in cognitive performance over time in Parkinson's patients, *R* ^2^ = 0.55, *F*(1,13) = 15.9, *P *=* *0.0016. This effect was also seen when we included unaffected participants (*R* ^2^=0.33, *F*(1,20) = 9.7, *P *=* *0.0054). This relationship was not driven by subtle differences in baseline MoCA, as there was no association between baseline MoCA and change in MoCA over time (*R* ^2^ = 0.07, *P *=* *0.34). Neither was there a relationship between dose of levodopa and performance in the skew task (*R* ^2^ = 0.10, *P *=* *0.19) or dose of levodopa and change in cognition over time (*R* ^2^ = 0.09, *P *=* *0.18).

### Neural correlates of skew image detection

**Figure 3:**
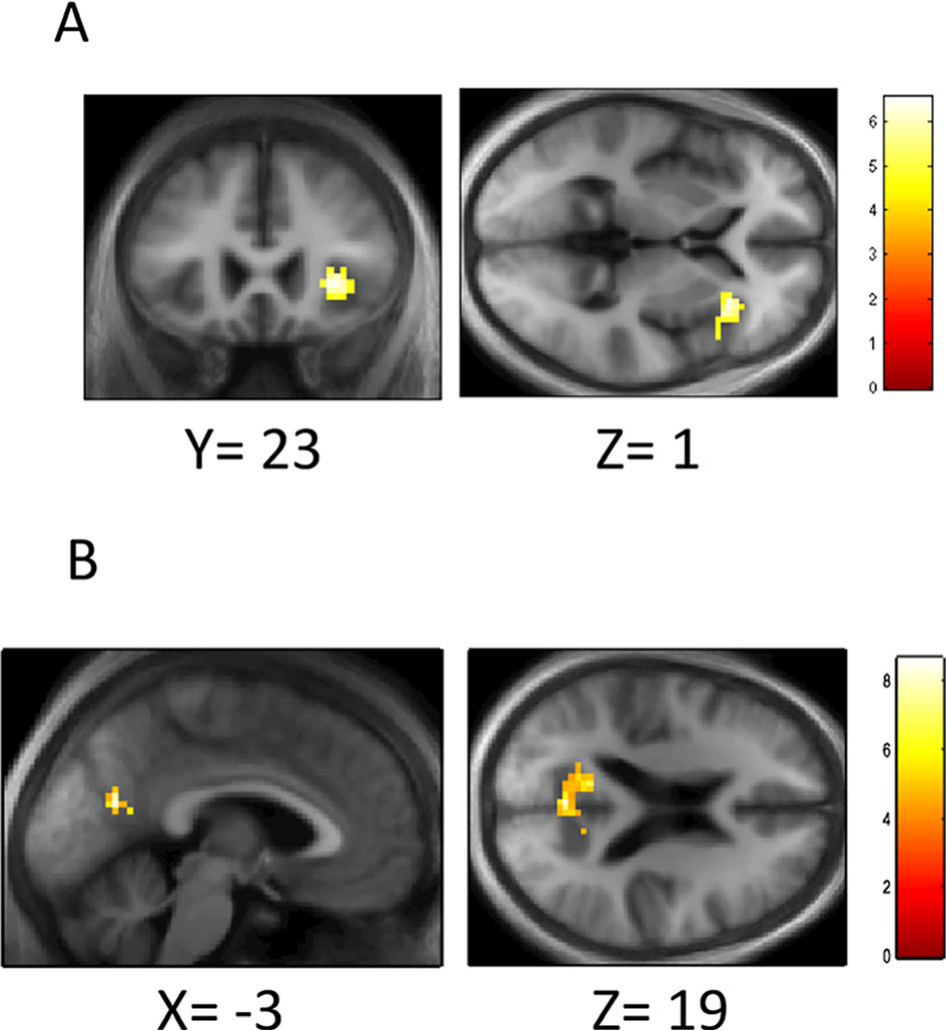
Neural correlates of skew performance. (A) SPM showing main effect of increasing difficulty across tasks in unaffected individuals, overlaid on the mean T1 image of all participants. Threshold for display < 0.001 uncorrected. (B) SPM showing the interaction of skew task and increasing difficulty in unaffected individuals, overlaid on the mean T1 image of all participants. Threshold for display < 0.001 uncorrected. Scale bars represent *T* value of coordinates.

Whole brain analysis of main effect of task (skew vs. noisy image) in unaffected controls did not reveal significant regions of activity at family‐wise error corrected levels. The main effect of difficulty revealed BOLD activations associated with higher difficulty in right insula, peak MNI coordinates [30, 23, 1], *k* = 50, *Z *=* *3.88, *P *=* *0.02 FWE‐corrected at cluster level, with a second peak within this at [48, 17, 1] *Z *=* *3.29 (Fig. 3A).

The interaction between task and increasing difficulty across all four levels of difficulty revealed BOLD differences within a region in medial parietal lobe corresponding to the PCC/precuneus, peak MNI coordinates [−3, −64, 19], *P *=* *0.001 FWE‐corrected at cluster level, *Z *=* *4.38, *k* = 124. Within this cluster, two other peaks were found: in the PCC/precuneus [−12, −58, 13], *Z *=* *4.30, and at [9, −58, 25], *Z *=* *4.25 (Fig. 3B). No other peaks survived correction for multiple comparisons.

We next inspected parameter estimates for each condition (four levels each for skew and noise) for each participant group (controls, high‐ and low‐performing Parkinson's patients) within the main cluster at the PCC/precuneus. This revealed a main effect of task (*F*(1,182) = 29.5, *P* < 0.0001). There was a strong interaction between the participant group (controls, high vs. low performers with Parkinson's disease) and task (skew vs. visual noise)) (*F*(2,182) = 12.3, *P *<* *0.0001), but no interaction between the group and difficulty or between the group, task, and difficulty. We also examined parameter estimates for Parkinson's participants only. This also revealed an interaction between the participant group (high vs. low performers with Parkinson's) and task (*F*(1,119) = 19.3, *P *<* *0.0001) and an interaction between the group and difficulty (*F*(3,119) = 3.0, *P* = 0.033, but no interaction between group, task, and difficulty.

**Figure 4:**
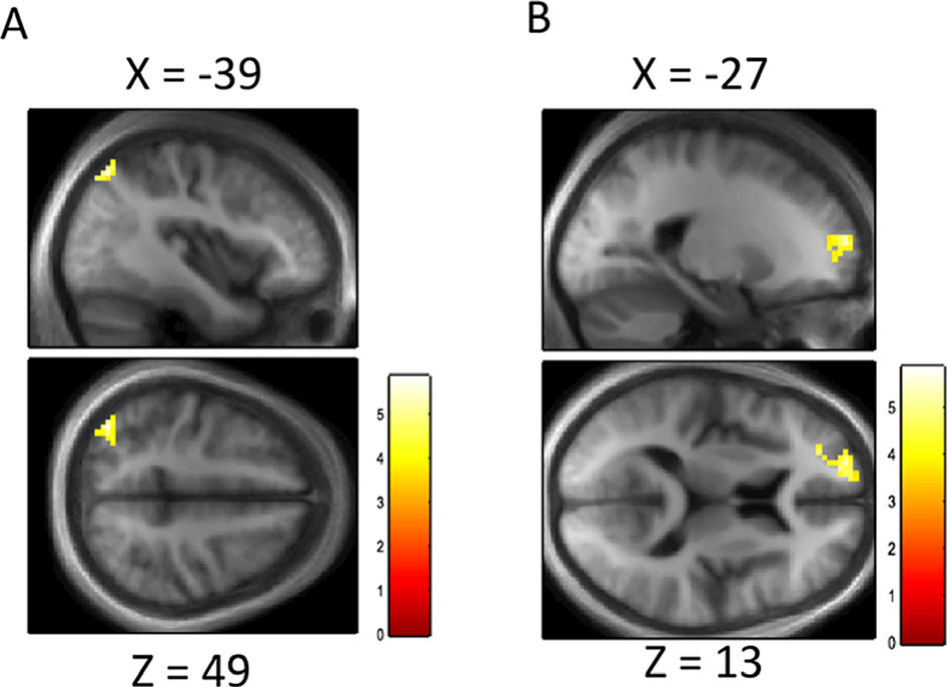
Neural correlates of skew performance in high‐ versus low‐performing Parkinson's patients. SPM showing greater BOLD activity in high‐ versus low‐performing Parkinson's patients in the left parietal (A) and prefrontal regions (B). Threshold for display < 0.001 uncorrected, with cluster level correction applied. Scale bar represents *T* value of coordinates.

Whole brain analysis of this interaction for each of the Parkinson's groups (high and low performers) did not reveal significant regions of activation, even at lower thresholds. However, when we compared brain activity between these two groups (high > low performers), for the interaction of skew/noise and difficulty, this revealed a cluster in left parietal lobe, close to the angular gyrus [−39, −67, 49], *P *=* *0.045 FWE‐corrected at cluster level, *Z *=* *4.27, *k* = 54, with a subpeak in the same cluster [−35, −76, 43], *Z *=* *4.23 and a further peak in the middle frontal gyrus [−27, 44, 13], *P *=* *0.037 FWE‐corrected, *Z = *3.98, *k* = 57 (Fig. 4A and B). These effects were not caused by artifacts linked with head movements. We examined head movements during scanning for each axis of movement (*x*,* y*,* z*, and pitch, roll and yaw) and did not see any differences in scan‐to‐scan head movements between high‐ and low‐performing patients with PD (see Table 3).

**Table 3:** Movement parameters in each dimension for participants at low risk versus high risk for dementia in Parkinson's disease

Axis	Low‐risk PD	High‐risk PD	*T*	*P*
*X* (SD) (mm)	0.051 (0.05)	0.025 (0.02)	1.5	0.18
*Y* (SD) (mm)	0.049 (0.007)	0.038 (0.02)	1.5	0.17
*Z* (SD) (mm)	0.16 (0.2)	0.091 (0.05)	1.0	0.34
Roll (SD) (deg)	0.091 (0.04)	0.076 (0.08)	0.57	0.57
Pitch (SD) (deg)	0.056 (0.03)	0.029 (0.02)	2.0	0.074
Yaw (SD) (deg)	0.052 (0.03)	0.031 (0.03)	1.6	0.13

Values are mean scan‐to‐scan movements in mm or degrees.

### Differences in functional connectivity in low‐ versus high‐performing Parkinson's patients

**Figure 5:**
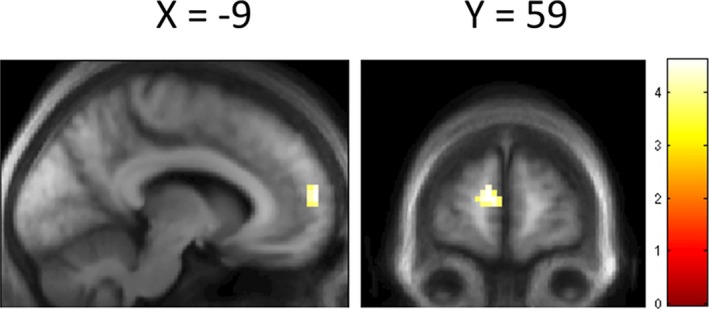
Psychophysiological interactions. SPM showing difference in functional connectivity to seed voxel identified in Figure 3B between high‐ and low‐performing patients with Parkinson's disease. Thresholded at < 0.001 uncorrected for display, with cluster level correction applied. Scale bar represents *T* value of coordinates.

Our behavioral finding of a positive association between performance in the skew task and change in cognition over time motivated us to test whether variation in skew detection is mediated via differences in functional connectivity between task‐specific areas and other regions across the whole brain. We used a psychophysiological interaction analysis between the PCC/precuneus and the rest of the whole brain to examine this. Our connectivity analysis showed that activity related to the skew task correlated positively with functional coupling between the PCC/precuneus and dorsomedial prefrontal cortex (dmPFC) in high‐performing, but not low‐performing Parkinson's patients, with a peak in the dmPFC, BA10 (Fig. 5, peak MNI coordinates [−9, 59, 10], *k* = 20, *Z *=* *3.67, *P *<* *0.001, uncorrected). This suggests that patients with Parkinson's disease with the earliest stages of cognitive involvement may show reduced functional connectivity between posterior and anterior nodes of the default mode network.

## Discussion

We aimed to identify the neural correlates of the early stages of cognitive change in Parkinson's disease. We show that performance in a visuoperceptual skew task correlates with change in cognition over time. We show that in unaffected individuals, the visuoperceptual skew task is mediated via activity in the PCC/precuneus, and that in people with Parkinson's who are worse at this task, activity in this region is reduced during task performance. Our functional connectivity analysis revealed that neural activity during this task correlated positively with the dmPFC, an anterior node of the default mode network that is beginning to be implicated in Parkinson's dementia.^11^

### Links to Parkinson's dementia

The importance of visuoperceptual deficits as an early indicator of Parkinson's dementia is evident from population^1^, ^22^ and FDG‐PET studies.^7^ Postmortem data show that occipital involvement is related to more rapid progression to Parkinson's dementia.^8^ Our previous behavioral work showed that visuoperceptual deficits are linked to poorer cognitive performance at baseline and to algorithmic scores predicting cognitive change.^12^

The neuroanatomical substrates of early cognitive changes in Parkinson's have not been previously described. Volumetric studies of gray matter change in Parkinson's do not show consistent regional thinning associated with cognitive change.^4^, ^6^, ^23^, ^24^ Recent cross‐sectional connectivity analyses in non‐demented Parkinson's disease show changes in occipital connections are linked to poorer cognitive performance.^25^ Our work now shows that change in the PCC/precuneus activity may be an important early indicator of future cognitive involvement in Parkinson's disease.

### Role of DMN in Parkinson's dementia

We identified changes in brain activity in the PCC/precuneus and reduced functional connectivity to the dmPFC during task performance. These regions form key nodes in the DMN, one of several brain networks identified using resting‐state fMRI^26^, ^27^ and thought to be involved in redirecting activity from internal to external goal‐directed processes.^28^ Changes in DMN functional connectivity may be associated with cognitive changes in Parkinson's disease. Decreased functional connectivity is seen within the medial temporal and bilateral inferior parietal cortex in Parkinson's compared with controls, with loss of connectivity correlated with cognitive performance.^10^, ^11^ Similarly, Yao^29^ found lower functional connectivity within the DMN in Parkinson's disease, including the PCC and precuneus. Other studies have shown similar findings.^30^, ^31^, ^32^

Reduced DMN activity may be linked with subtypes of Parkinson's at higher risk of dementia. For example, patients with the akinetic rigid form of Parkinson's disease show decreased DMN activity than those with the less severe tremor‐predominant form.^33^ Huang and co‐workers showed these differences in the left IPC and PCC^11^ and patients with Parkinson's with mild cognitive impairment similarly show reduced DMN activity.^34^

Deactivation in the DMN is seen in other dementias. Firbank and colleagues^35^ showed strong deactivations in the posterior DMN in Lewy Body and Parkinson's Dementias. Reduced DMN functional connectivity is well described in Alzheimer's disease.^36^ These changes are seen in MCI, prior to onset of Alzheimer's,^37^ in prodromal Alzheimer's disease^38^ and in carriers of genetic mutations linked to Alzheimer's.^39^, ^40^ Whether alterations in posterior nodes of the DMN are linked to cognitive deficits due to a key role in cognitive processing, or represents selective vulnerability due to high connectivity and metabolic demands is not yet known and whether the DMN is selectively affected ahead of other brain networks in Parkinson's dementia is not yet known and could be explored in future work.

### Limitations and future directions

There are some methodological considerations for this study. Although our findings survived statistical correction for multiple comparisons, our study included a relatively small number of subjects. The exploratory functional connectivity analyses were not corrected for multiple comparisons and will need to be replicated in larger cohorts. Not all patients with Parkinson's disease were available for follow‐up testing, although only a small proportion were lost to follow‐up, and our drop‐out rate of 21% is in line with other data series in similar patient groups.^41^, ^42^, ^43^ Our data include patients with varying disease duration which may influence performance and/or neural activity. Future studies could examine larger numbers, with a more detailed cognitive battery.

Although we showed some specificity of the skew task compared to the visual noise task, patients with Parkinson's disease also showed deficits in the visual noise task. This task probes earlier, lower level visual processing such as figure‐ground segregation. Our finding of deficits at these stages of visual processing is consistent with several other studies^44^, ^45^, ^46^, ^47^ showing that visual processing is affected throughout the visual processing axis in Parkinson's disease. Differences in the specificity for the skewed task may reflect heterogeneity in these relatively small patient samples.

It is also not possible to completely rule out that the effects seen at the PCC/precuneus were driven partly by residual differences in difficulty between the two tasks, although the form of the interactions detected there does not strongly suggest this.

Our participants were studied while on their dopaminergic medication and we did not find any relationship between levodopa equivalent dose and performance in the skew task or change in cognition over time. However, some studies suggest a link between dopamine levels and DMN activity in Parkinson's disease.^31^, ^48^ Future work could examine visuoperceptual performance at different stages of the medication cycle.

REM behavior sleep disorder (RBD) has also been linked to cognitive outcomes in Parkinson's disease^49^, ^50^, ^51^, ^52^ with a particular link between RBD and visuoperceptual deficits.^53^ We did not collect information on RBD in our participants, and this could be explored in future work.

These findings are of wider importance for patients with Parkinson's disease. By implicating posterior brain regions in the earliest stages of Parkinson's dementia, these may now be examined for potential to stratify patients for clinical trials of disease modifying interventions, or as potential biomarkers of progression. They also point to fundamental approaches that can be tested in future studies to identify mechanisms for selective vulnerability of particular brain regions for Parkinson's dementia.

In summary, we show that visuoperceptual deficits tested using a skew task predict worsening cognition in Parkinson's disease; that performance in this task is related to activity in the PCC/precuneus, with lower levels of activity in this region in poorer performing participants. Finally, we show that task‐related activity in the PCC/precuneus is associated with reduced functional connectivity to dmPFC, both regions implicated in brain networks linked with Parkinson's dementia. Our work thus reveals that visuoperceptual deficits, such as those detected with the skew task are fundamentally linked with critical regions affected at the earliest stages of Parkinson's dementia.

## Author Contributions

RSW conceptualized and designed the study, acquired and analyzed the data, and drafted the significant proportion of the manuscript. JSW analyzed the data and drafted the significant proportion of the manuscript. LAL acquired and analyzed the data. KP acquired and analyzed the data. RM acquired and analyzed the data. HRM conceptualized the study and drafted the significant proportion of the manuscript. GR conceptualized and designed the study, and drafted the significant proportion of the manuscript.

## Conflict of Interest

HRM reports personal fees from Teva, AbbVie, Boehringer Ingelheim, and GSK; RSW reports personal fees from GE.
